# Married Men Perceptions and Barriers to Participation in the Prevention of Mother-to-Child HIV Transmission Care in Osogbo, Nigeria

**DOI:** 10.1155/2014/680962

**Published:** 2014-02-19

**Authors:** Ademola L. Adelekan, Elizabeth R. Edoni, Oladipupo S. Olaleye

**Affiliations:** ^1^Department of Research and Reproductive Health, Public Health Promotion Alliance, Osogbo 3166, Nigeria; ^2^Department of Community Health Nursing, Niger-Delta University, Wilberforce Island 569108, Nigeria; ^3^Department of Health Promotion and Education, Faculty of Public Health, University of Ibadan, Ibadan 2000005, Nigeria

## Abstract

Men's role in HIV prevention is pivotal to changing the course of the epidemic. Men's barriers toward participation in Prevention of Mother-to-Child Transmission (PMTCT) have not been adequately documented. This study is therefore designed to determine men's level of awareness and barriers to their participation in PMTCT programmes in Osogbo, Nigeria. This study was a descriptive qualitative one that utilized Focus Group Discussion (FGD). One-hundred and sixty married men were selected by convenience sampling and interviewed. Data collected were analysed using content analysis technique. Demographic data were analysed using SPSS 15.0 software to generate frequency tables. Participants mean age was 31.9 ± 5.9 years. Many of the participants had heard about PMTCT and the majority agreed that it is good to accompany their wife to Antenatal Care (ANC) but only few had ever done so. Societal norms and cultural barriers were the leading identified barriers for male involvement in PMTCT programmes. The majority of the participant perceived it was a good idea to accompany their wife to antenatal care but putting this into practice was a problem due to societal norms and cultural barriers. Community sensitization programmes such as health education aimed at breaking cultural barriers should be instituted by government and nongovernmental agencies.

## 1. Introduction 

Globally, an estimated 35.3 (32.2–38.8) million people were living with HIV in 2012 [1]. The annual number of newly infected children in 2012 was 260 000 (230 000–320 000) in low- and middle-income countries, 35% lower than in 2009 [1]. Nearly all of these children acquired HIV through Mother-to-Child Transmission (MTCT) and ninety per cent of them live in sub-Saharan Africa [2]. These figures indicate not only the magnitude of the problem, but also the fact that pediatric HIV infections are numerous and worrisome [3]. In Nigeria, the estimated number of children under age 14 living with HIV is 360,000, and the estimated number of pregnant women living with HIV is 210,000 [4]. According to the latest Demographic and Health Survey, only 28 percent of women and 39 percent of men know that the risk of an HIV-positive pregnant woman transmitting the HIV virus to her unborn child can be reduced by taking certain drugs during pregnancy [5]. About 58 percent of pregnant women receive Antenatal Care (ANC) and the usage of Prevention of Mother-to-Child Transmission (PMTCT) services has only reached 12 percent [6]. In resource-limited settings, male partner involvement in antenatal HCT has been shown to increase uptake of interventions to reduce the risk of HIV transmission [7].

Historically, many Prevention of Mother-to-Child Transmission PMTCT programmes have organized their services as if potential clients were free to act independently. Thus, most awareness and implementation efforts related to family planning and HIV prevention and care have been directed primarily at women, disregarding the cultural and gender norms that may impact women's decision making regarding these issues [3]. The reality is that women's decision making about their pregnancies and health is deeply influenced by their partners, communities, and social norms and beliefs regarding HIV and AIDS [8]. The lack of male involvement in PMTCT consequently undermines the potential benefits of antenatal HIV preventive efforts [9], thus representing a missed opportunity to effectively prevent vertical HIV transmission. Male partners therefore seem to be the forgotten half of the equation [10]. Despite the potential benefits, male PMTCT involvement has some possible drawbacks. These include disruptions of family relationships, emotional and physical abuse for the spouses, loss of economic support, and blame and abandonment for the spouses [11–26].

Studies in Nigeria show that 74.6 percent of the men were not willing to participate in PMTCT programs by accompanying their wives to antenatal clinics due to being too busy (25.2 percent), cultural reasons (21.4 percent), and lack of knowledge of the importance of the program (21.4 percent), and 15.5 percent perceived ANC services as women's affairs [27]. In another study on community gatekeepers' awareness and perception of PMTCT services in Ibadan, Nigeria, it was reported that opinion leaders made up of religious leaders, heads of households, and leaders of community-based organizations had low knowledge of MTCT and PMTCT services, and community sensitization on the issue was inadequate [28]. Another study of male involvement in PMTCT services in Tanzania's Mbeya region revealed that although all the respondents generally accepted PMTCT interventions, barriers to involvement included lack of knowledge/information, no time, neglected importance, the services representing a “female responsibility,” or fear of HIV testing result [29].

Unfortunately in Nigeria, data on awareness and barriers to men's participation in PMTCT are generally scanty and the existing studies are rather similar in focus and limited in scope. This study was therefore designed to determine awareness and barriers to men's involvement in prevention of mother-to-child transmission of HIV programmes in Osogbo, Nigeria in order to inform programs aimed at enhancing male partner participation in PMTCT.

## 2. Materials and Methods

### 2.1. Study Design and Scope

This was a descriptive qualitative study that utilized Focus Group Discussions (FGDs). The scope of the study was limited to awareness, involvement, and barriers to participation in PMTCT programme among men.

### 2.2. Study Area

Osogbo is a city in Nigeria, the capital of Osun State. Osogbo has two Local Government Areas (LGAs), namely, Osogbo and Igboona LGAs. Yams, cassava, grain, cotton, and tobacco are grown. The majority of the population is from Yoruba ethnic group. Osogbo is the venue of the annual Osun-Osogbo festival along the River Osun. The festival is centred around the sacred grove of the river goddess Ọsun, which is a UNESCO World Heritage Site. Ladoke Akintola University of Technology (LAUTECH) teaching hospital is located in Osogbo, Nigeria. The hospital presently has a total of 290 beds and another set of 20 bedded facilities is being completed for private patients bringing the total beds to 310. Presently, the Hospital has an Out-patient Department (OPD) comprising of the general clinics and all special clinics. The Surgery Outpatient Department serves all the specialities of surgery, that is, general surgery, orthopaedic surgery, plastic surgery, urology, paediatric surgery, and neurosurgery.

### 2.3. Study Population

The study population are married men with inclusion criteria of at least 20 years of age, being married or having female partners within 18–45 years age group regardless of whether their wives had been involved in PMTCT service or not, and being a resident of Ladoke Akintola University Teaching Hospital catchment area in Osogbo.

### 2.4. Study Sample

A total of 160 participants were proportionally selected by convenience sampling. The researchers went round all the communities selected for the study and identified possible influencers who helped to mobilize married men in the communities. The detailed objectives of the study were explained to them and consenting eligible participants were recruited for the study. The recruited participants jointly agreed on the date and time for the discussion. The discussions were conducted in a suitable environment such as the community town hall, community leader compound and at the front of the participants' family house. Ten FGDs were conducted in each LGA and eight participants were recruited for each of the session.

### 2.5. Study Instrument

The FGD guide topics were derived from the literature together with input from health promotion specialists at the University of Ibadan, Nigeria. Some of the major questions on the study guide were as follows. Have you heard about the programme called PMTCT in this community/town? Are you aware of any woman who has been counselled and tested for HIV at PMTCT clinics? What are your sources of information on PMTCT? What do you understand by PMTCT programme? Do you think it is good to accompany your wife to ANC? Have you ever accompanied your wife to ANC? What do you think are your roles in your wife pregnancy? What are the barriers to male involvement in PMTCT?

### 2.6. Data Collection and Analysis

Data were collected between March and May, 2013 using FGD guide with open-ended questions. The guide was developed and pretested among married men in Ibadan. Ibadan shares similar characteristics with the study location. Before pretesting, the research team discussed the FGD guide to make sure that all involved in data collection understood and were acquainted with the guiding questions. The FGD guide was revised based on the issues identified by the team. The improved version of the FGD guide was used for pretesting exercise. Pretesting was conducted to check consistency and clarity of information being gathered by the FGD guide. No problems were identified with the revised FGD guide. Both pretesting and actual data collection were conducted in local language commonly spoken in the area (Yoruba).

The FGDs were moderated by one of the researchers with a research assistant experienced in qualitative methodology, who provided simultaneous translation from Yoruba (the local language) into English. The moderator controlled the sessions to make sure that everyone including the shy people participated in the discussions to avoid missing the distinctive perceptions of less talkative respondents. During the FGD sessions, probing questions were posed to seek clarification on issues and to encourage participants to give more insights in their responses. Efforts were made to give respondents sufficient discussion time on key issues. The discussions were conducted in a quiet space to avoid noise interference and interruptions and each session lasted about 40–69 minutes. The discussions were audiotaped with the consent of the participants. Tapes were transcribed verbatim in Yoruba and were subsequently translated into English. Spot checks of transcripts and translations were regularly conducted to ensure completeness of the transcription and accuracy of translation. The data were transcribed, sorted, categorized, and analyzed thematically. Demographic data were analysed using SPSS 15.0 software to generate frequency tables.

### 2.7. Ethical Considerations

Relevant information was verbally provided to the study participants before the FGDs to ensure that they make informed choices on whether to participate in the study or not. To ensure confidentiality, participants were informed that their names will not be recorded anywhere and that all the information gathered and subsequent reports would not refer to individual participants to ensure their confidentiality. No incentives were provided to the respondents as a way of motivating them to participate in the study. All the identified potential participants agreed to participate in the discussions and signed a consent form.

### 2.8. Limitation

Ascertaining the genuineness of responses provided by the study participants is a daunting challenge in research and this study has no exception. The spectrum of men used in this study may have had an influence on the accuracy of the data. If married men of pregnant women were used, the results may have been different.

## 3. Results and Discussion

### 3.1. Demographic Characteristics

A total of one-hundred and sixty married men participated in the FGDs and they were between 23 and 59 years with a mean age of 31.9 ± 5.9 years. Many (42.5%) were in the age group 28–35 years. The largest proportion (45.9%) of the participants had only Primary Education, Ordinary National Diploma/National Certificate of Education (10.8%) and 9.8% had Higher National Diploma/First degree. The majority (74.8%) of the participants were Muslims and 98.8% were of Yoruba tribe. Majority (97.5%) of participants were currently married while 2.5% were cohabiting. Only 3.2% of study participants had no children and 12.9% had two or more wives.

### 3.2. Awareness of Prevention of Mother-to-Child Transmission Services

Many of the participants had heard about the programme called the PMTCT and knew that pregnant women are being counselled and tested for HIV at PMTCT clinic. A participant from Okefia community said,

*I have heard of PMTCT before when my wife was pregnant last year.*

Another participant from Igboona community said;

*I heard of PMTCT from a friend who is a health worker in LAUTECH teaching hospital.*

This corroborates a research done in Malawi [30] and other similar findings from Nigeria [5, 7]. Participants were of the opinion that it is important for couples to get tested for HIV together with the intention of protecting their unborn child from HIV infection. A participant from Ajegunle community said,

*Mother who knows early in her pregnancy that she is HIV infected has more time to make important decisions. She and her health care provider will have more time to decide on effective ways to protect her health and prevent MTCT of HIV.*

Another participant from Oja-Oba community said,

*A woman can also take steps to prevent passing HIV to her partner.*

Sources of information about PMTCT among the participants were radio, television, health workers, and newspapers. Almost all the participants knew that PMTCT services were offered at Ladoke Akintola University Teaching Hospital, Osogbo.

Participants were further asked about their understanding of PMTCT programme. Majority of the participants said it was a service rendered to HIV positive pregnant women towards preventing HIV transmission from the mother to the child. A participant from Olaiya community specifically said,

*PMTCT services are special services for HIV pregnant women to let them understand various ways of HIV transmission and how their baby can be prevented from contracting HIV during pregnancy, delivery and after childbirth.*

Another participant from Okefia community said,

*This is a service render to pregnant women when they are positive to HIV so that their baby will not be infected with the virus.*

This finding is similar to a previous study [31]. The noted misconception among few participants was the perception that PMTCT services are for HIV infected women with high viral load in their body and that when this viral load is low, then there is no need for a woman to access this service.

### 3.3. Male Involvement in PMTCT

Participants were asked if they think it is good to accompany their wife to ANC. The majority of the participants agreed that it is good. This is in line with the findings by Nkuoh et al. [31]. Few of the participants added a condition that their presence must be needed at the clinic before they could accompany their wife. Participants believed they need to accompany their wife to ANC because she is carrying their pregnancy and also to be able to support her financially when the need arises in the clinic. Other reasons given by the participants were that accompanying their wives will help them to learn and increase their knowledge of ANC activities, show true love and keep the woman happy, encourage and support the woman during the stress and discomfort of pregnancy, and help both (the couple) to know their health status.

Only a few participants reported that they had ever accompanied their wife to ANC. A participant from Igboona community said,

*I have accompanied my wife to ANC many times because she gets sick a lot while pregnant.*

This is in accord with the findings by Nkuoh et al. [31]. Participants' reasons for never accompany, their wives to ANC were not having time due to work, the perception that ANC is for women only, that it is “not in our culture” for men to attend ANC with wife, and that the man will be perceived as a *“woman wrapper”* (a man who takes order from his wife) among his peers. A participant from Olaiya community said,

*Men have so many responsibilities towards meeting financial obligation of the family; so we cannot stay throughout the ANC with our wives because it takes long hours. But for me, what I do most times was to drop my wife at the clinic and leave for work and when she is through at the clinic, she will call and I will go and pick her.*

Another participant said,

*Men do not usually want to accompany their wives for antenatal visits because they do not want to be tested in the hospital.*

Another participant from Ajegunle community said,

*Not only that we do not want to be tested in the hospital but we also don't want to be told that we are positive to HIV.*

Participants were also asked about what they perceived to be their roles during their wife's pregnancy period. Many of the participants said that their roles are to pay the ANC/delivery fees and also care for their child or children if any. A participant from Igboona community said,

*I think our role is to pay the ANC bills and take care of children when necessary especially when the pregnancy is up to seven month.*

Another participant from Okefia community said,

*The role of a man is to pay the ANC bills and also get someone like my sister to assist her with domestic work.*

This is in accord with previous research done in Cameroon [31]. Participants were specifically asked if financial support was adequate support for men to provide to their pregnant wife. The majority of the participants responded that pregnant women need additional support on domestic work. A participant from Alekuwodo community said;

*I don't think financial support is what all women will need from us. I think our ordinary presence with them will go a long way towards making them happy which is good for their health.*

A contrary opinion from a participant from the same Alekuwodo community was that,

*If women can be provided with adequate financial support, they will be okay. At least they will use the money to get all they want.*

Participants were asked if they would support their wife in taking medication during pregnancy to prevent HIV transmission to her baby. Most of the participants indicated that they would support their wives to take the drugs to ensure that the baby is born free of HIV, especially if the drugs are free. A participant from Oja-Oba community said,

*“We would support her since the baby belongs to the husband”*

Another participant from Igboona community said;

*I will surely support her and also make sure I provide her with herbs because there are herbs women can also use to prevent this transmission.*

A contrary opinion was also recorded among few participants that they cannot support their wives in taking medication. A participant from Alekuwodo community said,

*I cannot support her at all because am scared of that drug and I will not be happy to see her taking it at all.*

Another participant from Ajegunle community said,

*I cannot support her but I will take her to my mother to help me provide support for her and am sure she will do that.*

Another reason why men would not support their wife was because of wrong perception towards antiretroviral drugs. A participant from Okefia community said;

*People say words that discourage HIV positive people from taking the drug especially those trado-medical people (traditional healers). They say it kills.*

### 3.4. Barriers to Male Involvement in PMTCT

Societal norms and cultural barriers were the leading identified barriers for male involvement in PMTCT programmes. A participant from Olaiya community specifically said,

*PMTCT clinics are made for women and children only.*

Another participant from Igboona community said,

*PMTCT is for women that are HIV positive.*

This is in line with most frequently reported barriers for male involvement in Antenatal Care identified across the different studies where males were of the perception that Antenatal Care was a woman's activity, and it was thus shameful for a man to be found in such settings [9, 29, 32–35]. Cultural barrier without any other external influence had been identified to demotivated men from attending ANC and getting involved in PMTCT [36].

Another identified barrier was lack of PMTCT knowledge among participants. The majority of the participants did not know the composition of this programme and thought that the only service rendered to infected pregnant mothers is to offer them medications. This is in accord with previous findings [28, 29, 37].

Few of the participants reported lack of finance as a reason why husbands do not support their wives. A participant from Dada estate said,

*Money is the major issue with most of us because when those nurses see that a woman comes to the clinic with her husband they start making financial demand with excuse that they want to use it to treat the woman and when such woman says she does not have money they will now tell her to go and collect it from her husband because the man is there.*

This corroborates the previous findings [31]. Long waiting times at ANC clinic were also cited by many of the participants as an obstacle to male involvement in PMTCT. Other barrier identified was that the timing of ANC/PMTCT activities was in conflict with men's normal daily activities. Other studies [29, 31, 33, 35, 38] have also noted.

## 4. Conclusion

Negative perceptions of male involvement in PMTCT were recorded among participants. This is attributed to the societal norms and cultural barriers. Interventions need to be implemented to change the negative perceptions and make men become effective educators and advocates for PMTCT. ANC services need to be made more male-friendly to encourage more men to attend with their spouses. The clinics should offer specific activities in which men can participate and try to adjust clinic hours to accommodate men's schedules. Health education aimed at breaking cultural barriers should also be instituted for men by government and nongovernmental organization as a possible way to address underlying gender norms and societal attitudes towards male involvement in PMTCT activities. Men could be brought to realize it is unacceptable to preserve outdated cultural norms at the risk of losing their lives and endangering infants.
